# Stress Adaptation and the Brainstem with Focus on Corticotropin-Releasing Hormone

**DOI:** 10.3390/ijms22169090

**Published:** 2021-08-23

**Authors:** Tiago Chaves, Csilla Lea Fazekas, Krisztina Horváth, Pedro Correia, Adrienn Szabó, Bibiána Török, Krisztina Bánrévi, Dóra Zelena

**Affiliations:** 1 Laboratory of Behavioural and Stress Studies, Institute of Experimental Medicine, 1083 Budapest, Hungary; tiago.chaves@koki.hu (T.C.); fazekas.csilla@koki.hu (C.L.F.); horvath.krisztina@koki.hu (K.H.); marques.correia.pedro@koki.hu (P.C.); szabo.adrienn@koki.hu (A.S.); torok.bibiana@koki.hu (B.T.); banrevi.krisztina@koki.hu (K.B.); 2 Janos Szentagothai School of Neurosciences, Semmelweis University, 1083 Budapest, Hungary; 3 Centre for Neuroscience, Szentágothai Research Centre, Institute of Physiology, Medical School, University of Pécs, 7624 Pécs, Hungary

**Keywords:** stress, brainstem, PVN, CRH, Barrington’s nucleus, inferior olivary complex

## Abstract

Stress adaptation is of utmost importance for the maintenance of homeostasis and, therefore, of life itself. The prevalence of stress-related disorders is increasing, emphasizing the importance of exploratory research on stress adaptation. Two major regulatory pathways exist: the hypothalamic–pituitary–adrenocortical axis and the sympathetic adrenomedullary axis. They act in unison, ensured by the enormous bidirectional connection between their centers, the paraventricular nucleus of the hypothalamus (PVN), and the brainstem monoaminergic cell groups, respectively. PVN and especially their corticotropin-releasing hormone (CRH) producing neurons are considered to be the centrum of stress regulation. However, the brainstem seems to be equally important. Therefore, we aimed to summarize the present knowledge on the role of classical neurotransmitters of the brainstem (GABA, glutamate as well as serotonin, noradrenaline, adrenaline, and dopamine) in stress adaptation. Neuropeptides, including CRH, might be co-localized in the brainstem nuclei. Here we focused on CRH as its role in stress regulation is well-known and widely accepted and other CRH neurons scattered along the brain may also complement the function of the PVN. Although CRH-positive cells are present on some parts of the brainstem, sometimes even in comparable amounts as in the PVN, not much is known about their contribution to stress adaptation. Based on the role of the Barrington’s nucleus in micturition and the inferior olivary complex in the regulation of fine motoric—as the main CRH-containing brainstem areas—we might assume that these areas regulate stress-induced urination and locomotion, respectively. Further studies are necessary for the field.

## 1. Introduction

Evolution enabled complex systems to develop intricate and dynamic biological functions not only capable to restore, but also maintain the equilibrium of their internal environment [1]. Any stimuli, either real or perceived (psychogenic), that threaten this balance can be defined as stressors. Psychogenic stressors are able to activate the adaptational systems even without a physiological stimulus [2]. These psychogenic stressors activate the brain in a top-down order and the descending information is summed up in the hypothalamus, more precisely in the paraventricular nucleus (PVN). However, the ascending information emerging from the internal milieu is first collected in the brainstem [3], then transmitted further to the PVN. Especially during systemic immune challenges, bidirectional communication exists between the PVN and the brainstem [4].

**Figure 1:**
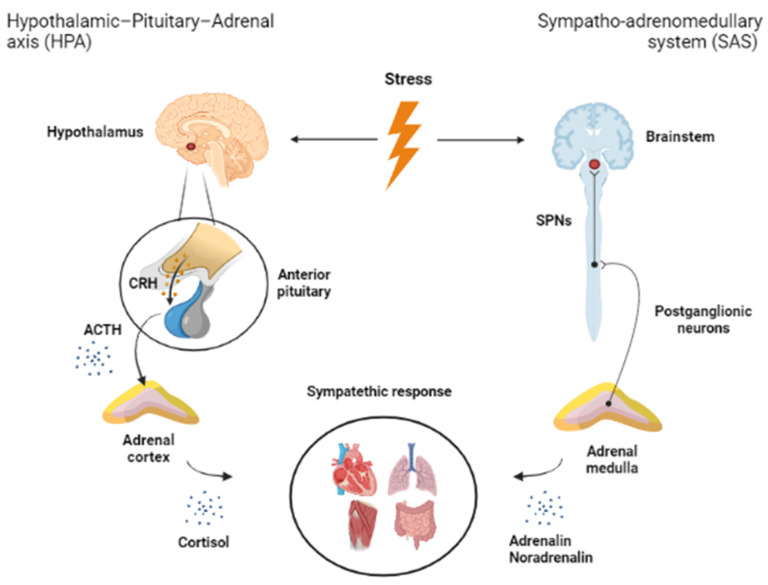
Main regulatory pathways of stress adaptation. There are two major limbs of stress adaptation: (1) the hypothalamic–pituitary–adrenocortical axis originating from the paraventricular nucleus of the hypothalamus, as well as (2) the sympatho–adrenomedullary system originating in the brainstem. Abbreviations: ACTH: adrenocorticotropin hormone, CRH: corticotropin-releasing hormone, SPN: sympathetic preganglionic neurons. This figure was created in BioRender.

Thus, external and internal stimuli activate the hypothalamic center of the hypothalamic–pituitary–adrenal (HPA) axis, the PVN, as a “final common pathway” [5] (Figure 1). Activation of the HPA axis is considered a hallmark of the stress response as it is responsible for the orchestration of the efficient usage of available energy in the body [6]. Neurosecretory neurons in the medial parvocellular region of the PVN release adrenocorticotropin (ACTH) secretagogues such as corticotropin-releasing hormone (CRH) and arginine vasopressin, and project to the blood vessels in the median eminence [7]. In response, the anterior pituitary secretes ACTH, which causes the adrenal cortex to synthesize and release glucocorticoids [8]. Negative feedback, a process in which end-products (glucocorticoids) limit their own release, is used to modulate the activity of the HPA axis due to the necessity to temporally constrain secretion [6]. Both inhibition and activation of glucocorticoid release is a well-coordinated process involving fast neuronal activation and timely inhibition. The inhibitory process can be fast and cease within minutes, resulting in the termination of PVN neural activity and ACTH release that characterizes an adaptative response to stress [9].

The central regulatory role of the hypothalamus is without doubt, but even the father of the stress concept, Hans Selye, recognized the importance of the sympatho–adrenomedullary system (SAS) during stress. According to his general adaptation syndrome theory, during the most acute phase of the stress response, there is a sympathetic activation leading to general changes in body homeostasis [10]. The ultimate goal is to avoid acute danger, which can be reached by a “fight or flight” response and translates to muscle activity [11]. To ensure optimal muscle function, blood supply has to be increased (tachycardia, hypertension), larger amounts of O_2_ (hyperpnea) and glucose (catabolic processes) should be provided, and the increased body temperature should be also normalized. These processes are regulated by catecholamines released from the sympathetic nerve endings (mainly noradrenaline (NA), also known as norepinephrine) or from the adrenal medulla (mainly adrenaline) (Figure 1). Both systems originate in the brainstem. Central pontine A5 noradrenergic neurons are directly connected to spinal sympathetic preganglionic neurons (SPNs), composing the base for SAS activity control [12]. The SPNs are located within the spinal cord. Their axons travel across the ventral horn, exit through the ventral roots, and reach sympathetic ganglia, where they form synapses with postganglionic neurons. The adrenal medulla is a special sympathetic ganglion, where the postsynaptic cells directly secret adrenaline into the bloodstream. Hence, the SNPs are the last control point for the brain on changes in sympathetic outflow [13].

The above-mentioned systems (HPA and SAS) are the basis of stress adaptation. This process is of utmost importance for the maintenance of homeostasis and, therefore, of life itself. Based upon Hans Selye’s theory, PVN, and especially its CRH neurons, are considered to be the centrum of stress regulation. However, as mentioned above, the brainstem seems to be equally important and other CRH neurons scattered all over the brain may also complement the PVN. Therefore, in this review, we will focus on the role of the brainstem in stress adaptation with special attention to CRH.

## 2. Acute or Chronic Stress

The outcome of neuronal responses to stressors is determined by their intensity, length, and frequency and is highly dependent on past stress experiences, manifested not only via behavioral patterns but also via endocrine changes [14,15].

Most of the studies focusing on stress regulatory pathways examined acute stressors due to less labor requirements and ethical considerations. However, chronic stress is implicated more in stress-related disorders. It is considered a major risk factor for almost every disorder, as it surpasses the regulatory capacity and adjustive resources of the organism and produces maladaptive responses [16]. Sometimes it is hard to properly distinguish between one and another, as recent work suggests that acute stress may also provoke long-lasting molecular and anatomical changes in the brain [17].

Nevertheless, in 1994, Greti Aguilera suggested that during chronic stress the activity of the HPA axis is maintained by vasopressin rather than CRH as it is less sensitive to the glucocorticoid feedback [18]. Subsequent studies (including experiments from Aguilera’s laboratory [19]) failed to confirm this idea [20,21].

Moreover, the general belief is that SAS does not contribute to chronic stress regulation (see its activation during the first phase of the general adaptation syndrome) [22]. However, several weeks of repeated immobilization results in elevated baseline catecholamine levels with reduced reactivity to a new homotypic challenge, but an exaggerated response to a heterotypic stimulus. Moreover, the level of the rate-limiting catecholamine synthesizing enzyme, tyrosine hydroxylase (TH), was increased in the noradrenergic brainstem neurons after repeated immobilization, electric foot-shock, and chronic social stress. Thus, adaptation to chronic stressors occurs in the SAS, too.

All in all, there is still a need to address the gap between acute and chronic stress-regulation [23].

## 3. The PVN-CRH Neurons and Stress Adaptation

CRH neurons are most abundant in the hypothalamic PVN region [24] and play a crucial role in the body’s adaptation to stress. They initiate hormonal cascades and coordinate stress-related behaviors through direct projections to limbic and autonomic brain systems [14,25,26].

Acute stress increases the steady-state *CRH* mRNA levels four hours after its initiation [27]. In the case of chronic, repeated stimuli desensitization of the ACTH response is observed, which also promotes the return of the transiently elevated CRH transcription to basal levels [28]. In contrast to the above-mentioned repeated homotypic stressor-induced inhibition, the PVN-CRH neuron activity is maintained or enhanced by heterotypic stressors [29]. Paradoxically, stress among certain conditions—such as lactation, or in the early stages of life, known as the stress hyporesponsive period—is characterized by decreased *CRH* expression and attenuated ACTH responses [30].

Daily fluctuation can be observed in the activity of PVN-CRH neurons which is most likely controlled by the suprachiasmatic nucleus of the hypothalamus (SCN) and is responsible for the development of the characteristic circadian pattern of HPA axis activity [31]. Indeed, the lowest level of glucocorticoids (and their upstream regulators) can be found during the beginning of resting phases (e.g., at the beginning of the night in humans, while in rodents at the beginning of the light period since they are nocturnal animals). Therefore, stress studies are generally conducted during this period allowing the identification of stressor-induced activations. Changing this long-standing practice should be considered, as stressors do not usually reach people during sleep, so studying stress processes in rodents during early light, thus in their sleeping period, may result in misleading information. However, the circadian rhythm may be upset by the sustained growth of glucocorticoids during chronic stress situations [32].

In relation to stress, CRH neurons, and their receptors also play a key role in the processes of learning and memory. Some studies have proved that a transient increase in CRH levels under acute stress promotes learning and memory development [33,34,35]. Acute stress results in *CRH* expression in hippocampal inhibitory interneurons which exert their effects through CRH receptor 1 (CRHR1), resulting in spine loss and dendritic remodeling of CA3 pyramidal neurons [36]. The glucocorticoid elevation induced apical dendritic retraction of CA3 pyramidal neurons may drive—among others—the deficit of spatial memory [37]. On the other hand, chronic stress reduces the dendritic complexity of CA3 neurons, which upsets the function of the HPA axis and leads to elevated glucocorticoid levels [38]. In mice, CRHR1 deficiency in the forebrain prevents the effects of chronic stress on CA3 dendritic length and complexity [26]. These findings confirmed the role of hippocampal CRH-CRHR1 signaling in modulating cognitive, structural, and molecular adaptations to chronic stress.

A close, albeit complex bidirectional relationship between central regulation of stress responses and energy homeostasis is assumed [39,40]. Depending on the duration and intensity of stress, the activity of pre-autonomic PVN neurons has an inhibitory effect on digestive processes [41]. Excessive food intake is thought to suppress, while prolonged hunger exacerbates stress responses, which can be explained by the fact that PVN-CRH neurons also receive inputs related to nutrition [42]. These observations confirmed that local hypothalamic mechanisms act at the level of CRH neurons and their afferent terminals are mutually integrated with the energy balance.

Food might also be considered, as reward and reward consumption allows rapid and strong inhibition of PVN-CRH neurons, resulting in a reduction of anxiety-like behavior and stress hormone surges [43]. Other authors also confirmed that fine foods reduce the HPA response to stress [44,45].

Considering pathological states, anxiety and depression are the best-known stress-related disorders. They are strongly connected to disturbed stress adaptation to chronic stimuli. Indeed, increased CRH content was found in the cerebrospinal fluid (CSF) of depressed patients [46]. Moreover, non-suppression in the dexamethasone test —testing altered feedback sensitivity—has been associated with an increased risk of suicide in depressed patients [47].

## 4. Function of Brainstem in Stress

The brainstem connects the cerebrum, cerebellum, and spinal cord [48]. Numerous internal and peripheral sensory information directly activate the brainstem, as it contains important areas of many vital functions for life, such as breathing, consciousness-sleep, blood pressure, and heart rate. All of these vital processes are adapting to the situation during stressor exposure. On the other hand, even external, physical, and psychological stressors activate the SAS, originating also in the brainstem and regulating the autonomic output in mammalian physiology [3]. Moreover, a bidirectional connection exists between the HPA and SAS [4].

### 4.1. Some Anatomical Considerations

The brainstem is composed of four sections in descending order: the diencephalon, midbrain, pons, and medulla oblongata [48]. Their cell bodies are arranged into important brain nuclei forming the grey matter. However, the origin of the white matter might be outside of the brainstem, as it contains many traversing pathways. Albeit, some of the white matter tract cell bodies are located within the brainstem as well and both receive and send axons to the periphery forming the so-called somatosensory pathways and the corticospinal tracts, respectively. Ten of the twelve cranial nerves arise from their respective brainstem nerve nuclei. The efferent and afferent connections with higher-order brain centers are also dense, and interaction with hormonal regulation may also occur here. For example, there is mounting evidence that sex hormones modulate autonomic physiology at the level of the brainstem [49].

### 4.2. Neurotransmitters

Classical neurotransmitters are stored in small synaptic vesicles (SSV) and released first after short action potential bursts [50,51]. The most important inhibitory neurotransmitter of the mammalian brain is gamma-aminobutyric acid (GABA) [52]. On the other hand, the main excitatory neurotransmitter, glutamate, is also abundant. Monoamine transmitters (serotonin or catecholamines such as NA, adrenaline, and dopamine (DA)) are also well-known, together with acetylcholine. They were first discovered at the periphery and till now their main role is supposed to be the sympathetic—parasympathetic regulation. They can be stored either in SSV or in large dense-core vesicles (LDCV) [53]. LDCV might be released extrasynaptically via the so-called non-synaptic [54] or volume transmission [55]. These small molecules might be part of normal cell metabolism, therefore, ubiquitous in all cells. Moreover, they are hard to detect, and thus, neurons operating with them are characterized by the specific vesicular transporters (vesicular GABA transporter, VGAT for GABAergic neurons; vesicular glutamate transporter, VGluT for glutamate; serotonin transporter, SERT for serotonin, etc.). Furthermore, their expression, detected at the mRNA level, can be followed in the cell bodies at the site of the origin of the efferents, while the proteins are present in axon-terminals, in the projection areas. These areas rarely overlap, but the GABAergic neurons are exceptions as they mostly form a local interneuron network [56].

Besides classical, small molecules all neurons contain several peptides, which are stored in LDCV and released upon stronger activation or extrasynaptically [51,55,57]. In relation to stress, CRH is the best-known neuropeptide transmitter. In fact, in the PVN, it is colocalized with glutamate characterized by VGluT2 [58]. In other brain areas, e.g., in the central amygdala (CeA) and bed nucleus of stria terminalis (BNST), it can be found in GABAergic neurons [59].

#### 4.2.1. Inhibition: GABA

GABA is synthesized from glutamate by decarboxylation (by the enzyme glutamate decarboxylase, GAD). It was first discovered in rotten pancreas in 1912, while in the 1950s its presence was confirmed in the mammalian brain with negligible amounts in other organs [60]. The majority of the synapses in the central nervous system (CNS) are GABAergic [61,62,63]. An interesting feature of these GABAergic neurons is their colocalization, e.g., with CRH. Some CRH-expressing, GABAergic, long-range-projecting neurons in the extended amygdala (i.e., BNST) innervate the ventral tegmental area (VTA) and may alter anxiety [64]. Paradoxically, GABA may also co-localize with glutamate [65]. Although the function of this colocalization is not clear, one might hypothesize that using multiple transmitters may serve to reduce the metabolic cost and errors of signaling. Moreover, during development GABAergic synapses might be stimulatory in contrast to their general inhibitory role [60]. One paper in 1997 showed that in SCN, GABA activated the neurons during the day and inhibited them at night [66]. However, this finding was not replicated, and it seems that the situation in the SCN is rather complex.

GABA may regulate the body’s physiological response to stress [67] by controlling the activation of CRH-releasing parvocellular neurons of the PVN [68]. In support, microinjections of GABA antagonists in the PVN promoted the activation of the HPA axis, resulting in an increase in the blood glucocorticoid levels [69]. On the other hand, microinfusions of GABA agonists into the PVN reduced the levels of stress hormones [70]. Due to this, systemic administration of GABA agonists, such as benzodiazepine, is beneficial for the treatment of stress-related disorders, e.g., anxiety [61,71,72]. Moreover, in the CSF, brain, and plasma of depressed patients, a low level of GABA was found, which was not normalized after treatment [73]. Thus, this low GABA function is proposed to be an inherited biological marker of vulnerability for the development of mood disorders. On the other hand, GABA_A_ receptor binding density was not altered in the locus coeruleus (LC), the main noradrenergic centrum with GABAergic innervation, suggesting that LC overactivity in depression may not be secondary to reduced GABAergic input to the LC [74].

Stress may also modulate the GABAergic system. A mild stressor, e.g., handling may increase, while stronger stimulus like foot-shock may decrease GABA binding-most probably reflecting changes in receptor numbers [75]. Additionally, formalin-induced acute stress resulted in a significant depletion of GABA levels in the lower brainstem as well as in the hypothalamus but not in the cortical areas [76]. On the other hand, increased motor and behavioral activity induced by a large dose of amphetamine markedly elevated the concentrations of GABA only in the major biogenic amine-containing brainstem cell groups (substantia nigra (SN), LC, and dorsal raphe (DRN)).

The brainstem GABAergic cells may also participate in stress regulation. One of the important regulatory sites might be the medullary raphe pallidus (RP or B1; see Section Serotonin) regulating sympathetic responses (e.g., heart rate or body temperature) via its premotor neurons [77]. RP neurons activate brown adipose tissue, the principal means for non-shivering thermogenesis, and cutaneous vasoconstriction in the tail, an important method of conserving body heat in rats. These sympathetic effects serve to maintain body temperature in a cold environment or to increase it during fever and are typically accompanied by tachycardia. Indeed, microinjection of the GABA_A_ receptor agonist muscimol into RP markedly attenuated stress-induced tachycardia. A similar response was seen after chemical stimulation of the dorsal hypothalamic area (DHA). Thus, the pathway from DHA neurons to sympathetic premotor neurons in the RP may constitute a key relay mediating the increase in heart rate seen during emotional stress. However, GABAergic neurotransmission within the nucleus tractus solitarius (NTS)—a sensory nucleus transmitting peripheral information to higher-order brain centers—may also regulate the heart rate during a threatening defense reaction [78].

#### 4.2.2. Excitation: Glutamate

Glutamatergic neurons are classified based on their VGluT content. The three subtypes discovered so far (VGluT1, 2, and 3) show complementary localization throughout the brain. The dominant isoform in the cortex is VGluT1, VGluT2 in the brainstem, while VGluT3 is expressed in certain subcortical nuclei [79].

Being the main excitatory neurotransmitter, the contribution of glutamate to stress adaptation is without doubt. Indeed, PVN-CRH neurons, the hypothalamic regulator of the HPA, contain VGluT2 [58]. Moreover, glutamate agonists (N-methyl-D-aspartate, (NMDA); and α-amino-3-hydroxy-5-methyl-4-isoxazolepropionic acid (AMPA)) may stimulate the HPA through the PVN [80], while systemic administration of glutamate antagonists (NMDA antagonist: MK-801; AMPA antagonist: GYKI52466) may inhibit stressor-induced glucocorticoid release [81]. Moreover, repeated stress increased spontaneous excitatory activity in the PVN-containing brain slices [82].

Brainstem glutamatergic neurons may directly influence the activity of the PVN. Indeed, the periaqueductal gray matter (PAG), zona incerta, subparafascicular nucleus, and lateral parabrachial nucleus send VGluT2 rich projections to the PVN, while raphe nuclei, and medullary portions are less implicated in this respect [83]. Glucagon-like peptide 1 positive cells in the NTS also express VGluT2 and project to the PVN [84] and their activation result in stress-related hormonal [85,86,87] and behavioral changes [88].

It was also shown that different stressors may influence the brainstem glutamatergic system. Mice that underwent chronic mild stress had increased glutamate levels in the raphe nuclei, but chronic social defeat stress did not yield the same result [89]. Another study showed that rostral medullary raphe nuclei (B1 and B3, see Section Serotonin) express c-Fos—a marker of neuronal activation [90]—after social defeat, a model for psychosocial stress [91]. Furthermore, it was also found that VGluT2-positive dorsomedial hypothalamic cells innervate VGluT3-positive B1 and B3 nuclei and elicit thermogenesis through sympathetic activation [92,93]. Rats submitted to chronic stress showed the classical signs of depressive-like behavior—a stress-related psychopathology—along with alterations in glutamate receptor (AMPA and metabotropic receptors) mRNA levels in the brainstem [94]. It is worth mentioning that acute stressor-induced changes were different from the effects of 2 weeks of repeated stimuli.

Stress may also affect the brainstem reward circuit potentiating substance abuse: social defeat stress facilitated NMDA receptor-mediated long-term potentiation (LTP)—a model of synaptic learning—in the VTA as well as the cocaine-induced place preference [95]. On the other hand, inhibiting glutamatergic neurotransmission (via AMPA and NMDA receptors) in the VTA of stressed rats rescued them from cocaine addiction [96]. These changes seem to be glucocorticoid dependent, as glutamatergic excitatory synapses are strengthened in midbrain dopaminergic neurons after glucocorticoid receptor activation via dexamethasone [97].

#### 4.2.3. Monoamines

##### Serotonin

The majority of serotonin (also known as 5-hydroxytryptamine, 5-HT)-producing neurons can be found in the raphe nuclei of the brainstem. There are nine distinct nuclei: raphe pallidus (B1 or RP), raphe obscurus (B2), raphe magnus (B3), certain cells of the reticular formation (B4), raphe pontine (B5), dorsal raphe (B6-7 or DRN), and median raphe (B8-9 or MRN) [98,99]. Based on their antero-posterior localization, they show distinct projection patterns: the midbrain raphe nuclei (DRN and MRN) mainly innervate the forebrain, while the nuclei in the pons (B4-5) and medulla (B1-3) project caudally to the brainstem and spinal cord (see review [98]). Although, for example, the B3 nucleus shows prominent direct efferent projections to the PVN, the majority of these innervations are not serotoninergic [100]. These nuclei also receive a wide range of afferents from the forebrain, among others dense innervation comes from hypothalamic and limbic areas [101,102,103,104]. Indeed, direct CRHergic innervation from the PVN might exist as the raphe nuclei express CRH receptors, and the activity of these receptors modulate the serotonergic system [105], and behavior [106,107]. However, this modulation was found to be relevant only in the case of external stressors such as shock [108], restraint [109], tail suspension, or forced swim stress [107].

The role of the serotoninergic system in stress and especially in depression—one of the best know stress-related psychopathologies—has been implicated on numerous occasions. For example, RP regulates sympathetic responses on the heart and body temperature. Significant c-Fos activity was measured here after cold swim stress [110]. Moreover, restraint stress-activated nesfatin-1 positive cells in the rostral RP [111]. Cardiovascular responses are mediated via the activation of 5-HT_1A_ somatodendritic autoreceptors in the RP: inhibition by the 5-HT_1A_ receptor agonist 8-hydroxy-2-tetraline hydrobromide (8-OH-DPAT) resulted in the abolishment of heart rate and blood pressure increment upon various stressors [112]. These were mainly acute effects, but 5-HT is also implicated in long-term stressor-induced changes. For example, prenatal stress resulted in an anxious behavioral phenotype in adult rats, which was accompanied by decreased *5-HT_1A_ receptor* mRNA levels in the raphe nuclei and susceptibility to drug abuse [113].

The inhibition of the rostral RP via the GABA_A_ receptor agonist muscimol resulted in the absence of tachycardia and hyperthermia upon audiogenic stress [114]. The opposite is true as well: disinhibition of the nucleus via GABA_A_ receptor antagonist bicuculline increased the heart rate, arterial pressure, and cardiac sympathetic nerve activity in anesthetized rats [115]. Thus, GABA innervation—most probably through local interneurons—may influence the activity of the serotoninergic neurons.

Similar to the RP, the DRN also showed c-Fos activity upon cold swim stress [110] and conditioned fear stress [116]. The DRN was already activated after a single injection of anesthetic, while the B2—a well-known centrum of chemoreception [117] —and B5 showed enhanced c-Fos positivity only upon moderate stressors [118]. Apparently, there is a sex difference in the DRN-CRHR1 activity: antagonizing the receptor resulted in a diminished hormonal response, a high number of c-Fos positive cells, and less anxious behavior in males, while neurons from female mice showed reduced excitability upon CRH binding in brain slices [119]. Interestingly, the DRN also regulates maternal care via CRH receptors [120].

The DRN and MRN seem to play a role in different aspects of stress reactivity. The mRNA levels of the tryptophane hydroxylase-synthesizing enzyme of serotonin already increased after a single restraint stress in the MRN, while for a similar stimulation in the DRN repeated stress protocols were needed [121]. However, chronic social defeat led to different results: serotonin-system-related mRNA levels (tryptophane hydroxylase, SERT, monoamine-oxidase, 5-HT_1A_ receptor) were all decreased in the midbrain raphe nuclei (DRN and MRN) even after 2 weeks in correlation with increased anxiety- and depressive-like behavior [122]. Electric stimulation of the MRN reduced stressor-induced plasma corticosterone levels [123]. Moreover, stress adaptation is mediated via 5-HT_7_ receptor activation in the MRN [124].

A further connection between stress and the brainstems serotoninergic system is supported by the monoamine theory of depression and the effectiveness of serotonin reuptake inhibitors (SSRI) in depression [125]. However, later theories suggested that the main effect of—at least some—SSRI antidepressants might be via increased neuroplasticity [126].

Nevertheless, based upon the above-mentioned experiments, we can conclude that the serotoninergic system actively regulates stress responses, but different anatomical subdivisions might have different functions. For example, the medullary and pons nuclei regulate the sympathetic answers to stress, while the midbrain nuclei organize the behavioral aspects.

##### Catecholamines

Catecholamine-containing nuclei in the brainstem represent the main source of catecholamines in the CNS. Neurons belonging to these nuclei produce and release either NA, DA, or adrenaline [127]. NA can be detected upon the presence of TH—the first and rate-limiting enzyme of catecholamine biosynthesis—as well as dopamine beta-hydroxylase (DBH)—the final enzyme in converting DA to NA—but the absence of phenylethanolamine N-methyltransferase (PNMT), the characteristic enzyme for adrenaline synthesis [53].

If these nuclei in the so-called lateral zone of the brainstem contain catecholamines, the neuronal phenotype is labeled with the letter “A”, and this is currently the case for NE or DA, while adrenaline-releasing neurons were later distinguished with the letter “C” (in contrast to serotonin-producing nuclei with the letter “B”) [127].

From a phylogenic perspective, the mesencephalic DA system, represented by A8 (retrorubral field), A9 (SN pars compacta), and A10 (VTA) nuclei, is probably the most ancient component of the reticular formation. However, all catecholaminergic brainstem nuclei are highly conserved structures during the evolution of the CNS, and are involved in the regulation of basic activities such as breathing, blood circulation, sleep-waking cycle, and motor control [127]. Most of them are also implicated in stress. Different subdivisions might have slightly different roles [128], supporting the theory that the brain categorizes stressors by a specific activation pattern [129].

##### Catecholamines: Noradrenaline (NA) and Locus Coeruleus (LC)

NA is the major neurotransmitter in the sympathetic nerve endings at the periphery. However, it can be also found in the CNS, e.g., in the brainstem nucleus LC (also known as A6) [53]. The LC is localized in the fourth ventricle base and is known for its vast and divergent efferent system, whose noradrenergic fibers reach nearly the entire neuroaxis. On the other hand, CRH innervation (in co-localization with excitatory neurotransmitters coming mainly from the PVN) directly controls LC neuronal excitability and activity via CRHR1 receptors. The connection between LC and PVN—the centrum of the HPA axis—is bidirectional, as massive noradrenergic innervation reaches the CRH positive cells of the PVN [130]. Of particular interest are the afferents expressing CRH from the CeA, which are thought to activate the LC to engage cognitive processes in response to environmental stressors, thus, has been conceptualized as the cognitive limb of the stress response [131,132]. Indeed, LC is also part of the central ‘stress circuitry’, because robust activation of the LC has been reported after divergent stressor exposure in experimental animals.

NA is also connected to depression since, in humans, the NA content of depressed subjects was changed in their LC [74]. Moreover, lower levels of an NA metabolite in the CSF was associated with suicide risk [47].

##### Catecholamines: Dopamine (DA)

In recent years, the dopaminergic system has been investigated with great interest in regard to stress regulation [133,134,135]. DA is a catecholamine neurotransmitter in the mammalian brain, responsible for controlling—among others—locomotion, cognition, emotion, positive reinforcement, and endocrine function [136]. Most importantly, changes in the brain levels of DA have been associated with stress-related psychopathologies, such as post-traumatic stress disorders (PTSD), substance abuse disorder, and psychotic disorder [137,138].

The dopaminergic transmission originates from the brainstem and is organized in four major neural pathways; the nigrostriatal, the mesolimbic, the mesocortical, and the tuberoinfundibular pathways [139]. (1) The nigrostriatal pathway stems from the SN pars compacta and ends in the dorsal striatum. This pathway is responsible for the regulation of locomotion—the degeneration of this pathway is known to cause Parkinson’s disease [140]. It also contributes to feeding behavior [141]. (2) The mesolimbic pathway starts in the VTA and ends at the nucleus accumbens. This pathway is associated with motivation, reward, and pleasure [142], although there is growing evidence that it is also implicated in stress-related disorders, such as anxiety and depression [143]. (3) The mesocortical pathway starts from the VTA and projects to the prefrontal cortex, and it is associated with emotion and cognitive functions, such as attention and planning [144,145]. (4) Lastly, the tuberoinfundibular pathway projects from the arcuate nucleus of the hypothalamus to the median eminence, and it is involved in the constant, tonic inhibition of secretion of the prolactin hormone [146,147,148].

The dopaminergic response to an acute stressor seems to be remarkably dependent on the stress source. Response to physical stressors is usually linked to an increase in DA activity in the dorsal striatum, whereas psychological stressors seem to enhance medial prefrontal cortex DA content [149]. Many studies have shown an increase in the DA release when the animals were facing aversive stimuli. This suggests that DA is likely to be involved not only in the processes underlying a positive reward but also in aversive events [150,151]. It has been reported that repeated restraint stress in animals changes the way the mesolimbic DA system responds to a stressor and that repeated stressors, such as foot-shocks, increase the self-administration of psychostimulants, thus, indicating a possible direct relationship between the DA system and the HPA axis [137,152].

As for the endocrine regulation, the DA-regulated prolactin—besides its well-known function in lactation—is also a stress hormone [81]. Divergent stressors may induce hyperprolactinemia and evidence shows that this stressor-induced prolactin secretion plays a significant role in the development of stress-induced pathology, including intestinal and tracheal epithelial barrier dysfunction, cardiac dysfunction as peripartum cardiomyopathy, and emotional problems [153]. However, prolactin secretion during stress may have an important physiological role in maintaining homeostasis within the immune system [154]. Indeed, in contrast to the immunosuppressive role of glucocorticoids, prolactin stimulates the immune system. Moreover, prolactin may also play an important role in maintaining metabolic homeostasis [155]. Stress-level of prolactin improved insulin sensitivity and decreased adipose tissue dysfunction in obese rodents and humans.

On the other hand, prolactin can influence the HPA axis at several points, among others, by increasing the secretion of ACTH and the sensitivity of the adrenal cortex to ACTH [156]. Prolactin may also directly induce adrenal steroidogenesis and catecholamine synthesis.

All in all, brainstem dopaminergic cells are deeply implicated in stress processes both among physiological as well as pathological conditions.

## 5. CRH Cells in the Brainstem

Based upon the importance of CRH as well as the brainstem in stress regulation we hypothesized that CRH synthesized locally in the brainstem might also be important in stress adaptation. In support, not only PVN but also BNST and CeA CRH-positive cells react to stressors suggesting a ubiquitous role of CRH in stress response [157].

However, detecting and visualizing CRH-positive neurons in the brain is challenging due to its low basal level in the cell bodies and the absence of highly sensitive CRH antibodies. Therefore, earlier studies used in situ hybridization to visualize CRH-producing cells at the mRNA level. In the mice brainstem, the highest *CRH* mRNA density was present in the Barrington’s nucleus (B) as well as in the inferior olivary complex (IO) [158]. Lower levels were detected in the tegmental (TRN) and parabrachial nuclei (PB) as well as in ventrolateral medulla and medial vestibular nucleus. Scattered cells were also present in the PAG, external cuneal nucleus (ECN) and in the raphe region.

Several attempts were also made to detect the CRH protein. For instance, immunohistochemistry can be used after invasive tissue exploration. However, the disadvantage of this procedure is the relatively reduced number of detectable neurons [159]. With colchicine treatment, a more accurate CRH cell distribution can be monitored throughout the brain, as it results in peptide accumulation in the soma by blocking the axonal transport of the neurons. By this method, the presence of additional CRH neurons in PB was found beside the intensive labeling of B and IO regions [160] (Table 1). However, despite the better immunoreactivity, it is doubtful if this technique is able to detect physiologically relevant CRH positivity. This notion is supported by the fact that CRH synthesis may occur in neurons upon stimulation/injuries [161,162], thus, the colchicine treatment itself may induce ectopic *CRH* expression.

A recently developed genetic technique allows the expression of a fluorophore under specific, e.g., CRH promoter. Thus, the direct visualization of CRH neurons is possible without influencing the physical state of the animals or the need to use any other supplementary detection methods. Furthermore, while immunohistochemical identification of CRH neurons provides a current protein content above a detection threshold at an appointed time, genetic technique tracks cellular expression of *CRH* over the lifespan of a mouse [163]. Previous studies using green fluorescent protein (GFP) under the CRH promoter identified once again the IO and B as highly CRH-positive areas of the brainstem both at the level of GFP as well as based upon their *CRH* mRNA content measured by in situ hybridization [164] (Table 1). In fact, the intensity of the signal here was comparable with the ones measured in the PVN and in the piriform cortex. Much lower CRH levels were found in other, previously mentioned brainstem nuclei including the raphe magnus. Another study using Venus fluorophore [165] or enhanced yellow fluorescent protein (EYFP) [166] also confirmed the *CRH* expression in the B, as one of the main extrahypothalamic CRH sources, as well as in the IO. Scattered cells were found in other, previously mentioned areas including raphe nucleus (especially MRN [165]). To further confirm the presence of CRH in the brainstem nuclei we crossbred CRH-Cre mice with dtTomato reporter mice containing the fluorophore between two loxP loci (DIO: double inverted open reading frame). In this case, the CRH-positive cells of the offspring (*Crh-IRES-Cre;Ai9*) were marked with a red fluorescent protein. With this technique, we confirmed the presence of CRH in previously mentioned brainstem areas (Table 1). The highest density of TdTomato expressing cells was found in the IO and B regions. Besides that, a considerable amount of CRH-positive cells was located in the TRN, PB, PAG, and ECN as well as in the medial vestibular nucleus, mesencephalic reticular formation, and pontine gray. The fluorescent signal of CRH neurons was also perceived in raphe nuclei, nucleus incertus, and interpeduncular nucleus.

The above-mentioned data arose from the examination of mice, but besides fundamental similarities, some species differences may also exist. After colchicine treatment, rats showed more intense labeling and in them, the dorsal lateral tegmental area contained the most CRH-positive cells, which was immunonegative in mice [160]. However, IO and B contained a considerable amount of CRH also in rats. In frog (*Xenopus laevis*), CRH was found in the tegmental area as well as in the LC [167]. In humans, DRN and MRN showed enhanced CRH immunopositivity in depressed subjects, however, this was attributed to enhanced innervation rather than an increase in local synthesis [168].

Overall, there are several CRH expressing neuron populations in the brainstem, but the exact role of these cells is still unknown

## 6. Assumed Function of Each CRH-Containing Brainstem Nuclei in Stress

The functioning of many brainstem nuclei can be related to the regulation of stress (see earlier in relation to neurotransmitters), but the role of locally synthesized CRH in stress regulation is highly neglected. We tried to summarize the available knowledge, but further studies are required to confirm that not only does CRH innervation coming from the PVN and/or CeA provide a connection to stress, but locally produced CRH is also important in stress adaptation.

**Figure 2:**
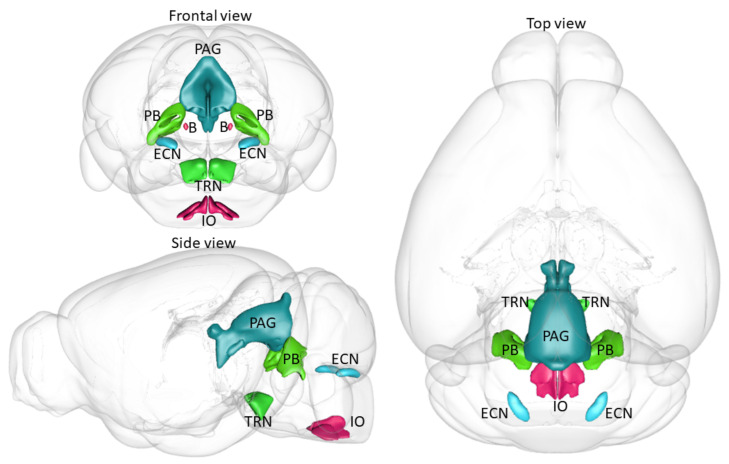
Mouse brain areas containing a significant amount of CRH-producing cells. The figure was created with the help of scalablebrainatlas.incf.org. Red: high CRH density; B: Barrington’s nucleus; IO: inferior olivary complex. Green: repeatedly confirmed moderate CRH density; TRN: tegmental reticular nucleus, PB: parabrachial nucleus. Blue: moderate CRH density; ECN: external cuneate nucleus, PAG: periaqueductal grey.

As one of the main CRH sources in the brainstem, Barrington’s nucleus (B) (Table 1, Figure 2) regulates micturition with the help of its CRH-positive neurons [169]. We might assume that B is implicated in the stress response as well based on its direct connection with the PVN [170]. In support of this hypothesis, c-Fos positivity was increased in this area after numerous stressor exposure. Moreover, some stressors, such as water avoidance stress, may increase the urinary frequency [171] and social stress-induced long-lasting voiding dysfunction [172], further connecting B to stress. In relation, upregulation of the CRH in B accompanied the social stress-induced urinary retention and a CRHR1 antagonist prevented this abnormal urodynamics [173]. This mechanism may underlie the development of stress-induced bladder disorders.

The other highly CRH-positive area of the brainstem is the inferior olivary complex (IO) (Table 1, Figure 2). As this area has a tight connection with the cerebellum, it has an important role in the regulation of motor coordination and motor learning [174]. Therefore, it is possible that CRH in this brain area is also involved in the fine-tuning of motor functioning. Even less is known about the role of this nucleus in stress. So far only morphine-induced c-Fos activation of this area was described [175].

A smaller amount of CRH can be found in the tegmental reticular nucleus (TRN) (Table 1, Figure 2) with an even smaller amount in the mesencephalic reticular formation. They are also connected to the cerebellum regulating eye movement participating in posture regulation [176,177], but with an unknown role in stress. There is no functional information about the CRH neurons of the parabrachial nucleus (PB) either (Table 1, Figure 2). This brain area transmits sensory information to forebrain structures such as itch, pain, or taste [178], but none of these were associated with its CRH-positive neurons. The external cuneate nucleus (ECN) [179] (Table 1, Figure 2), as well as pontine gray, may also contribute to the regulation of motor skills [180].

In contrast, the moderately CRH-positive PAG (Table 1, Figure 2) was shown to regulate restraint stress-induced anxiety-like behavior in Wistar rats, tested by nonselective CRH receptor antagonist (alpha-helical CRH9-41) microinjections [181]. This research group previously investigated the effect of CRH administration directly into the dorsal PAG and found an anxiogenic effect in the elevated plus maze test [182]. However, in this experimental design, a CRH receptor antagonist had no effect on anxiety-like behavior, questioning the results of the agonist administration.

The previously mentioned raphe nuclei are of utmost importance in stress regulation. However, their role is connected to their serotonin content (see Section Serotonin). They contain only a small amount of CRH, but we must acknowledge that in the MRN the serotonin-positive cells are also sparse, only 8.5% [183]. Thus, we cannot close out an important role of the raphe-CRH cells in stress regulation. Raphe-CRH interaction was studied in connection to freezing behavior during a fear conditioning paradigm—a model of learning and memory as well as fear [184]. Injecting both CRHR1 and CRHR2 antagonists into MRN decreased freezing during fear recall. Moreover, when a nonselective CRH receptor antagonist was administrated into the DRN before an inescapable shock, it prevented a behavioral response to uncontrollable stress. Furthermore, direct administration of CRH mimicked the effects of inescapable shock, however, only when it was administered at a high dose and into the caudal part of DRN [185]. We must acknowledge that CRH administration, as well as CRH receptor manipulation in these nuclei, model the effect of innervation rather than the role of locally produced CRH.

In relation to other CRH-containing brainstem nuclei, the nucleus incertus is also important in fear memory formation [186]. Its GABAergic cells contain CRH receptors and project to stress response regulatory centers such as the PVN [187]. In agreement, intracerebroventricular CRH injection increased its c-Fos immunoreactivity [188]. Again, these results suggest the importance of CRH innervation rather than the role of locally synthesized neuropeptides.

A study on guinea pigs suggested that vestibular stress influences HPA axis activity: after unilateral vestibular deafferentation increased night salivary cortisol level was measured [189]. The Palkovits laboratory explored the connections between vestibular nuclei and PVN neurons with the help of retrograde tracing. They found vestibulo-paraventricular polysynaptic, but not monosynaptic pathways, which might play an important role in vestibular stress-induced HPA axis activation [190]. None of the related research revealed the identity of the participating neurotransmitters or neuropeptides, therefore, we cannot conclude on the role of vestibular CRH, either.

In relation to the interpeduncular nucleus, a study published in 2020 found that the interpeduncular-ventral hippocampal serotonergic pathway is important in stress coping [191], but there is no information if CRH contributes to this phenomenon.

The vagus nuclei (NTS/dorsal vagal complex) may also contain scattered CRH-positive cells [160] and might deeply influence HPA axis activity being a key regulatory node for coordination of acute and chronic stress [192].

## 7. Conclusions

Despite the main focus on PVN and HPA in stress adaptation, the role of the brainstem should not be neglected. It may influence the HPA axis via a bidirectional connection with the PVN. Their different neurotransmitters might have divergent roles in fine-tuning the stress response. Beside classical neurotransmitters (GABA, glutamate, as well as serotonin, NA, DA, or adrenaline), several neuropeptides might be co-localized in the brainstem nuclei. Here, we focused on CRH as its role in stress regulation is well-known and widely accepted. Indeed, the CRH brain network seems to be stress-sensitive, forming an ancient, unified stress regulatory system [157]. Although CRH-positive cells are present on some parts of the brainstem, sometimes even in amounts comparable to the PVN [164], not much is known about their contribution to stress adaptation. Based on the role of the Barrington’s nucleus in micturition and the inferior olivary complex in the regulation of fine motoric—as the main CRH-containing brainstem areas—we might assume that these areas regulate stress-induced urination and locomotion, respectively. Further studies are necessary for the field.

## Figures and Tables

**Table 1:** Brainstem regions of the mouse that contains CRH-positive neurons.

	Density of CRH + Neurons.				
	Wang et al. 2011	Alon et al. 2009	Kono et al. 2016	Peng et al. 2017	Recent Results
Method	Colchicin	CRH-GFP	CRH-Venus	CRH-EYFP	CRH-Cre × DIO-dtTomato
Inferior olivary complex (IO)	++	++++	++++	+++++	+++
Barrington’s nucleus (B)	++	++++	++++	++++	++
Tegmental reticular nucleus (TRN)	-	++	++	+++	++
Parabrachial nuclei (PB)	++	++	+	+	+
Periaqueductal gray (PAG)	-	++	+	++	+
External cunate nucleus (ECN)	-		+	+++	+
Raphe nucleus	-	+	++ (MRN)	+ (DRN)	+
Nucleus incertus	+		+	+	+
Mesencephalic reticular formation	-	+	+	+	+
Interpeduncular nucleus			+	+	+
Pontine gray				+++	+
Medial vestibular nucleus	-		+	++	+
A11		+			
Vagus nuclei (NTS, motor)	++				

The areas are ranked according to the density of the cells during our experiment. Abbreviations, not given in the Table: CRH: corticotropin-releasing hormone, DIO: double inverted open reading frame, DRN: dorsal raphe nucleus, EYFP: enhanced yellow fluorescent protein, GFP: green fluorescent protein, MRN: median raphe nucleus, NTS: nucleus tractus solitarius, (In our recent experiment: ”+” = number of CRH + cells/mm^2^ < 5; ”++ ” = number of CRH + cells/mm^2^ < 5–10; ”+++ ” = number of CRH + cells/mm^2^ > 15; in other experiments more ”+” indicate stronger relative expression, while ”-” indicate a value bellow the detection limit).
